# Gamified Assessment of Medication Literacy in School-Aged Children: Instrument Development and Validation Study

**DOI:** 10.2196/95114

**Published:** 2026-10-02

**Authors:** Qingqing Liu, Lihua Cheng, Guanfu Liu, Jieli Peng, Zhiyu Wang

**Affiliations:** 1 Children’s Hospital of Nanjing Medical University Nanjing China; 2 Independent Researcher Nanjing China; 3 Nanjing Gulou No.1 Central Primary School Nanjing China

**Keywords:** children, gamified assessment, medication literacy, methodological comparison, psychometric validation, responsiveness

## Abstract

**Background:**

Assessing medication literacy in children is essential for evaluating school-based health education, yet traditional measurement tools often fail to engage children, compromising data quality. Gamified assessments offer an alternative, but their psychometric properties, comparability to conventional formats, and responsiveness to intervention effects require systematic evaluation.

**Objective:**

This study developed and evaluated a gamified assessment tool for measuring medication literacy across 4 domains (knowledge, attitude, perceived behavioral control, and behavioral intention) in elementary school children through 3 sequential phases: psychometric validation, methodological comparison with a paper-based questionnaire, and application to interventions.

**Methods:**

Three independent cohorts were recruited from a single elementary school in Nanjing, China, using cluster random sampling of intact classes (grades 4-6). We adapted the CHERRIES (Checklist for Reporting Results of Internet E-Surveys) framework. Phase 1 (40/81, 49.4% female) includes content validity via 2 rounds of expert review, construct validity using Rasch analysis, and 1-month test-retest reliability. Phase 2 (42/85, 49.4% female) includes a randomized crossover design (10-minute washout), equivalence assessed with an intraclass correlation coefficient (ICC), Bland-Altman analysis, decision consistency, and superiority using the chi-square test (data quality) and 2-tailed independent *t* tests (enjoyment and preference on 5-point Likert scales). Phase 3 (41/85, 48.2% female) includes a single-group pre-post design, with responsiveness evaluated using generalized linear mixed models (GLMMs) with random intercepts. Analyses used R software with an α of .05.

**Results:**

Phase 1 had perfect content validity (scale-level content validity index/average [S-CVI/Ave]=1.00), and the Rasch analysis supported unidimensionality (eigenvalues 2.14-2.25, ratios 0.34-0.40) and item fit (mean infit/outfit mean-square [MNSQ] 0.83-1.23). The test-retest ICCs were 0.76 to 0.92 (95% CI 0.64-0.95). Phase 2 equivalence was confirmed (ICC 0.94-0.99, 95% CI 0.90-0.99; κ=0.87-0.97). Validity rates were 100% (42/42, 95% CI 91.6%-100%) for gamified vs 90.7% (39/43, 95% CI 77.4%-96.9%) for paper (*P*=.13). The gamified tool yielded higher enjoyment (mean 4.26, SD 0.94 vs mean 2.46, SD 0.85; Cohen *d*=2.00, 95% CI 1.46-2.55; *P*<.001) and preference (mean 4.29, SD 0.83 vs mean 2.82, SD 0.97; Cohen *d*=1.62, 95% CI 1.11-2.13; *P*<.001). Phase 3 GLMMs showed time effects for attitude (*β*=1.74, 95% CI 1.25-2.22; *P*<.001), perceived behavioral control (*β*=2.86, 95% CI 2.18-3.53; *P*<.001), behavioral intention (*β*=2.33, 95% CI 1.51-3.15; *P*<.001), and knowledge (incidence rate ratio [IRR]=1.52, 95% CI 1.22-1.89; *P*<.001); time × class interactions were nonsignificant (all *P*≥.19), indicating consistent changes across classes. Effect sizes were moderate to large (Cohen *d*=0.70-1.53, 95% CI 0.36-1.97).

**Conclusions:**

This study develops and validates a gamified assessment tool for children’s medication literacy across 3 independent phases, including a randomized crossover comparison with a paper-based format. Unlike child health measures relying on adult proxy reports or adapted adult scales, our child-centered tool was directly validated with students and designed to enhance engagement. The psychometrically sound tool achieves measurement equivalence while offering a pediatric health-promotion outcome measure. The tool can support school-based medication literacy programs and advance health equity by detecting consistent improvements across diverse classrooms.

## Introduction

The “Healthy China” initiative and related national policies [1-3] emphasize the critical role of individuals as the primary agents of their own health, advocating for the public to adopt appropriate health perspectives and enhance health literacy, which is defined as the ability to access, understand, appraise, and use information and services in ways that promote and maintain good health and well-being [4], a key determinant of health equity as recognized by the World Health Organization [5]. However, current health literacy research, including associations with medication adherence, self-medication behavior, and other health-related outcomes, has predominantly focused on adult populations [6-13], while largely overlooking children and adolescents as active participants in their own health development [14]. Even studies related to child health often target adult caregivers rather than children themselves [15-20].

A fundamental challenge in advancing child health literacy research is the scarcity of valid and engaging assessment tools tailored to children’s cognitive and developmental levels [14,21]. Existing tools, often adapted from adult questionnaires that typically assess functional skills such as reading prescription labels, understanding medication instructions, and calculating dosages [22], fail to fully engage children and may be susceptible to inattention and social desirability biases, thereby limiting the accurate assessment of intervention impacts and the understanding of children’s health competencies [23].

This gap is especially pressing given that schools are widely recognized as crucial settings for fostering health literacy in children [5,24-27], and there is growing policy emphasis on integrating health care professionals into school-based health initiatives for children [1,3]. Without child-centered measurement tools, however, the effectiveness of these efforts cannot be accurately evaluated. In this context, gamified assessment emerges as a methodological solution to the limitations of traditional tools [28]. By incorporating game elements, it aims to reduce assessment biases and enhance participant motivation [29-31], thereby potentially improving the validity and reliability of data collected from children.

While the knowledge-attitude-practice (KAP) model offers a basic pathway from knowledge to behavior, its application in children is limited by their dependent health behaviors and the strong influence of caregivers. Merely improving knowledge and attitudes may not sufficiently enhance behavioral autonomy, and tracking actual behavior change remains challenging. In contrast, the theory of planned behavior (TPB) emphasizes behavioral intention—shaped by attitudes, subjective norms, and perceived behavioral control (PBC)—as a direct predictor of behavior. Subjective norms are inherently present in the school setting, arising from teacher expectations and peer behavior [32]. Because these norms are relatively stable over the short study period and are not directly targeted by the intervention, we did not include them as measured variables in our assessment tool. Instead, we focused on the modifiable individual-level components of the TPB (attitudes, PBC, and behavioral intention) in our assessment tool. Enhancing PBC can strengthen children’s autonomy. Moreover, behavioral intention can be assessed through contextualized scenarios, making the evaluation of health behavior change more feasible. The tool, which integrates the KAP framework with the key TPB components—knowledge, attitudes, PBC, and behavioral intention—translates these constructs into engaging game mechanics to enhance children’s engagement and support the evaluation of intervention outcomes.

Within child health literacy, one critical dimension warrants focused attention: medication literacy—a foundational health skill encompassing the knowledge, skills, and confidence required for the safe and appropriate use of medications [33]. Pharmacotherapy is the most common intervention for childhood illnesses, yet medication poisoning consistently ranks as a leading cause of pediatric poisoning [17]. This underscores the need to equip children with medication literacy—a competency that remains largely overlooked, and for which developmentally appropriate assessment tools are virtually absent.

Therefore, to address this methodological gap, this study aimed to develop and preliminarily validate a novel, theory-informed gamified assessment tool for measuring medication literacy in school-aged children, and to evaluate its psychometric properties, methodological performance, and responsiveness, thereby providing an effective means to evaluate future child-focused health promotion initiatives.

## Methods

### Ethical Considerations

The study protocol was approved by the Medical Ethics Committee of the Children’s Hospital Affiliated with Nanjing Medical University (approval number 202505033-1). Administrative permission was obtained from the participating school prior to study initiation. Informed consent was obtained from all parents or legal guardians. Child assent was also secured verbally from each participant immediately before activities. To protect privacy and adhere to data minimization principles, only essential nonidentifiable variables were retained for analysis: gender, class, and grade. Student IDs were temporarily used solely to match paired responses across the 3 phases and were permanently deleted upon completion of matching, replaced with anonymized participant codes. All analyses were conducted using this deidentified dataset. No compensation was provided to participants or their families for taking part in the study. The manuscript and supplementary materials contain no images that could identify individual participants. All game screenshots are generic and do not include any personal information.

### Conditions and Design

This methodological study comprised 3 independent, sequential phases (Figure 1) to develop, validate, and demonstrate the application of a novel gamified assessment tool for child medication literacy.

**Figure 1:**
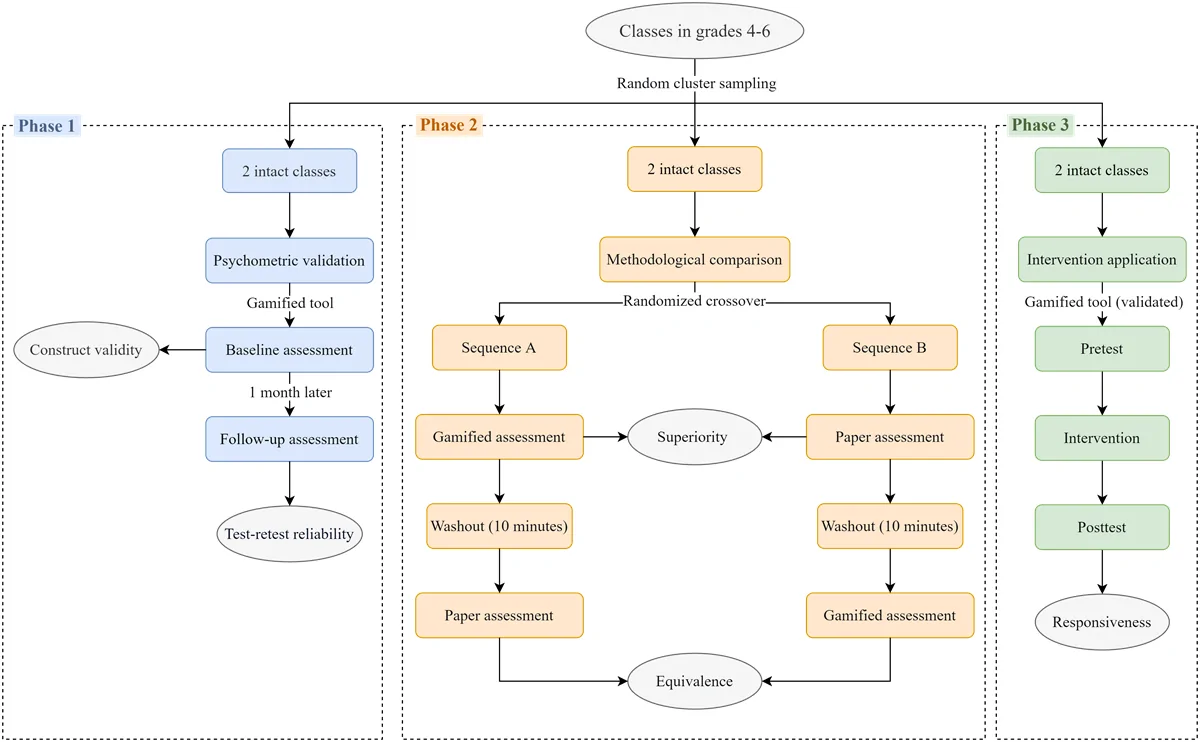
Study design overview of the 3 independent phases: psychometric validation (phase 1), methodological comparison (randomized crossover, phase 2), and intervention application (pre-post design, phase 3) in elementary school children (grades 4-6) in Nanjing, China (2025). Phase 1 establishes psychometric properties using the gamified tool. Phase 2 uses a randomized crossover design to compare the gamified tool with a traditional paper-based questionnaire. Phase 3 applies the validated tool in a longitudinal intervention study.

Phase 1 aimed to establish the tool’s psychometric properties using an independent sample, without any prior intervention, including content validity through a 2-round expert review, construct validity via Rasch analysis, and test-retest reliability with a 1-month interval.

Phase 2 used a randomized crossover design with a separate sample, also without any prior intervention, to methodologically compare the new tool against a traditional paper-based questionnaire, testing its equivalence and superiority (data quality and participant experience). Participants were randomly allocated to 1 of 2 administration sequences and completed 2 assessments: sequence A (gamified → paper-based) and sequence B (paper-based → gamified), with a 10-minute washout period between sessions, which corresponded to the regular break between 2 class sessions, chosen to minimize disruption to the school schedule while providing a brief period to reduce short-term memory of responses.

Phase 3 applied the validated tool in a longitudinal, independent pre-post study with a sample distinct from those in prior phases to assess its practical responsiveness (generalized linear mixed models [GLMMs]) in detecting changes following a school-based medication literacy intervention. We implemented a compact pre-post assessment using the validated gamified tool, with participants completing the assessment immediately before and after the intervention, to maximize sensitivity to change while minimizing confounding. The intervention was designed as a single 40-minute in-person session, which included a 20-minute interactive educational component delivered by trained pharmacists, with the remaining time used for the pretest and posttest. To ensure age-appropriateness, we adapted core components from the *Chinese Citizens’ Health Literacy—Basic Knowledge and Skills* (2024), focusing on medication literacy relevant to general child health rather than adult-centric topics. The selected components centered on three core principles: (1) distinguishing dietary supplements from medicines, (2) rational medication use principles, and (3) science-based and rational health care practices. To translate these principles into an engaging format, interactive educational material titled “The Great Journey of a Little Medicine” was created, which adopts a first-person narrative from the perspective of a medicine describing its metabolic journey. The storyline translates these principles into four specific educational objectives: (1) dietary supplements are not medicine substitutes, (2) preference for oral over infusion, (3) no unilateral discontinuation for side effects, and (4) no alarm for drug-induced urine discoloration. To ensure consistency, the session was delivered in a standardized manner by the same trained pharmacist, following a three-part format: (1) engaging storytelling centered on the narrative, enhanced with animated demonstrations, (2) explicit emphasis on core medication literacy points, and (3) interactive question-and-answer and experience-sharing to facilitate immediate application, clarify concepts, and promote personal engagement.

This phased, independent-sample design ensured that psychometric properties and methodological advantages were established prior to and uncontaminated by intervention effects, aligning with best practices for novel measurement tool validation.

### Inclusion and Exclusion

The study included elementary school students in grades 4 to 6. Students present on the scheduled assessment days with parental consent and child assent were included; no additional exclusion criteria were applied.

### Participant Characteristics

Participants in psychometric validation were recruited from 2 classes in grades 4 and 6. The total sample for this phase consisted of 81 students (40/81, 49.4% female). Methodological comparison involved a separate cohort from 2 classes in grades 5 and 6. A total of 85 students (42/85, 49.4% female) participated in the randomized crossover study. Intervention application recruited a third independent cohort from 2 classes in grades 5 and 6, comprising 85 students (41/85, 48.2% female).

### Sampling Procedures

The study was conducted in a single public elementary school in Nanjing, China. Data collection for the 3 phases took place between September and December 2025. Using cluster random sampling, distinct intact classes from grades 4 to 6 were randomly selected as separate, nonoverlapping cohorts for each phase. The overall study targeted grades 4 to 6, as children in these grades (approximate age 9-12 years) have the cognitive and literacy skills needed to understand medication-related concepts and complete the gamified assessment independently. This choice was supported by the elementary school teachers who participated in our content validity review. We used grade level rather than chronological age because age within the same grade is inconsistent (birthdays occur at different times, and some families use the traditional Chinese nominal age system). In contrast, grade provides a relatively uniform educational background, allowing students to be reasonably treated as a single group for analysis.

### Sample Size, Power, and Precision

The sample size was determined based on the practical capacity of the health care professional team and the feasibility of implementation within the school’s routine. Given a maximum class size of 40 to 45 students, and to match the health care professionals’ workload, minimize disruption to normal school schedules, and align with available devices, we planned to recruit 2 intact classes for each phase of the study. This provided sufficient participants for each phase while allowing for occasional absences. Because of the pilot and methodological nature of the study, formal a priori power calculations were not performed. Instead, sample size adequacy was assessed against methodological literature. For Rasch analysis, simulation studies have shown that item calibration remains stable even with small samples (n≤50) [34]; our phase 1 sample (n=81) exceeds this threshold, supporting reliable parameter estimation. For the GLMMs with clustered data, methodological research indicates that GLMMs remain a consistent analytical approach when the number of clusters is small [35], and using appropriate small-sample corrections can control type I error rates [36]. In our study, the within-class sample size (n=40-45 per class) together with the Satterthwaite approximation (default in the *lmerTest* package, detailed in the Analytic Strategy section) supports the reliability of the GLMM results.

### Instrumentation

We developed a novel gamified digital assessment, which was structured around the integrated KAP-TPB framework and comprised 4 core dimensions: knowledge, attitudes, PBC, and behavioral intention. Detailed descriptions of the scoring rules and measurement procedures are provided in the Measures and Covariates section.

### Measures and Covariates

The game content was directly derived from the key learning objectives of the planned medication literacy intervention. Although our assessment employed gamified interactions rather than traditional paper-based items, we adapted the CHERRIES (Checklist for Reporting Results of Internet E-Surveys) framework [37] to report on user recruitment, engagement metrics, and data completeness, as these principles remain applicable to digital survey modalities.

To reduce abstraction and enhance measurability, we designed the game to generate contextualized, ordered responses rather than rely on abstract self-report. Each dimension was operationalized through a distinct game mechanic (Figure 2): knowledge was assessed via a 5-item “target practice” game where children selected answers by clicking on targets. Each item had 1 correct option; an “I do not know” choice was included to minimize guessing. Responses were scored 1 (correct) or 0 (incorrect/I do not know). Attitude was measured using a 4-item “block-hitting” game. Children jumped a character to hit blocks corresponding to their agreement with statements on a 5-point Likert scale (1=strongly disagree to 5=strongly agree), with both positively and negatively worded items. PBC was evaluated through a 4-item “speech preparation” drag-and-drop task. For each topic, children selected from 2 correct arguments (+1 each), 2 incorrect arguments (–1 each), and an “I do not know” option (0). The total score (–2 to 2) was then mapped onto a 5-point Likert scale. Behavioral intention was gauged via 4 interactive “scenario simulation” role plays. Children faced medication-related dilemmas in a continuous storyline that provides a realistic and coherent decision-making context and chose among 5 predefined behavioral responses, each prescored 1 to 5 based on theoretical alignment with rational medication literacy.

**Figure 2:**
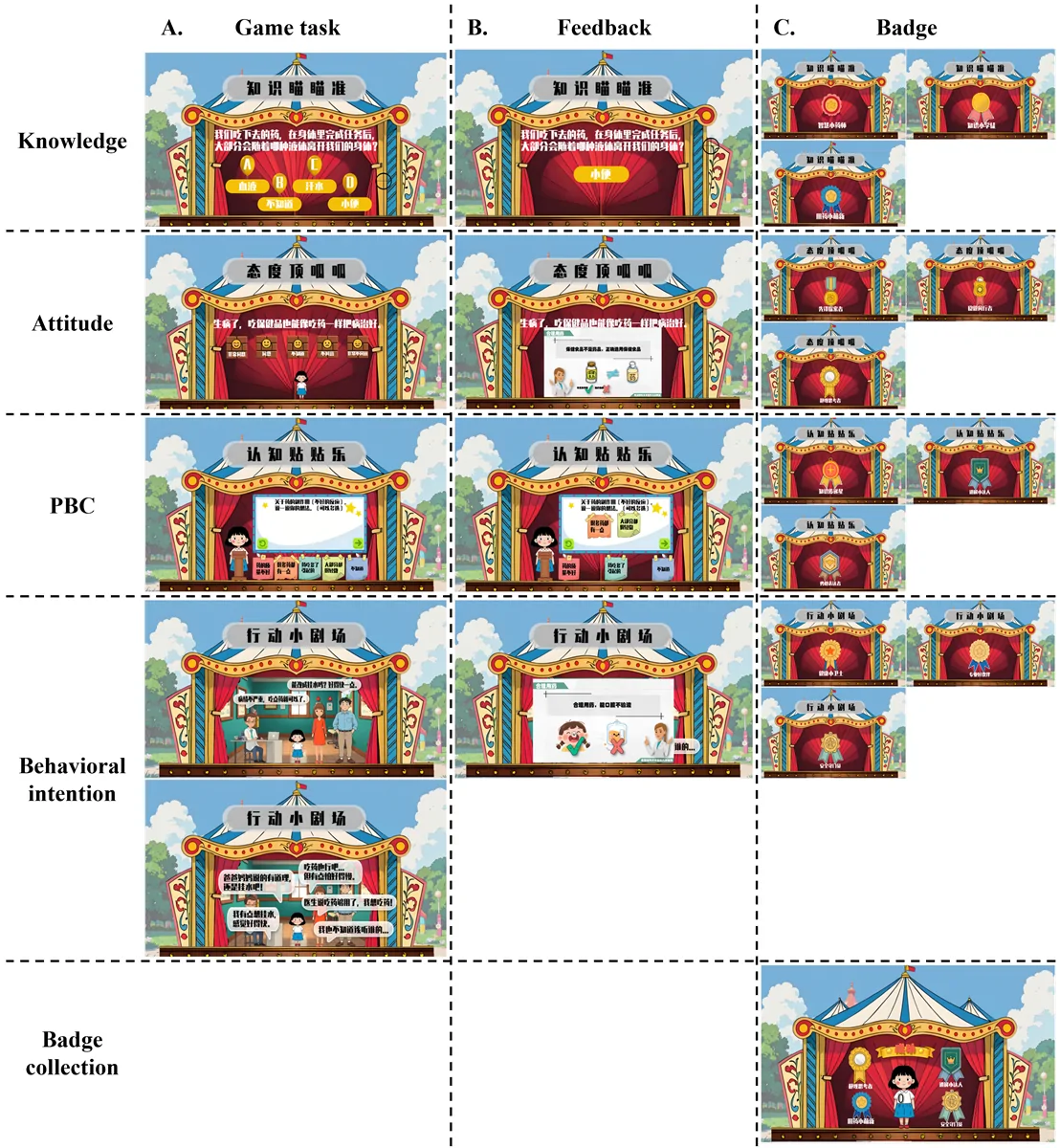
Interface of the gamified medication literacy assessment tool used in all phases among elementary school children (grades 4-6) in Nanjing, China (2025). The figure illustrates the 3 core components of the gamified assessment for each dimension: (A) the game task interface, (B) the feedback screen following task completion, and (C) the badge awarded based on performance. Rows 1 to 4 correspond to the 4 assessed dimensions: knowledge, attitude, perceived behavioral control (PBC), and behavioral intention. The final row displays the badge collection screen, where all earned badges are aggregated upon completion of all tasks. The interface is presented in Chinese, the language of instruction for participants. Table S1 in Multimedia Appendix 1 provides English translations of all texts shown here, along with item-level justifications and educational objectives, and badge English translations and design rationale are provided in Table S2 in Multimedia Appendix 1.

To mitigate testing anxiety and reduce social desirability bias, we replaced traditional numerical scores with a performance-based digital badge system. Participants earned a unique badge upon completing each game dimension. Crucially, badges were not titled with explicit performance tiers (eg, no “gold” or “silver”) to minimize peer comparison. The complete badge system, including score ranges and design rationale, is presented in Table S2 in Multimedia Appendix 1. All badges were aggregated and displayed on a final “badge collection” screen (Figure 2). This approach was designed to elicit more authentic, low-pressure assessments of children’s knowledge, attitudes, PBC, and behavioral intention.

To transform the assessment into a learning-reinforcement tool, we provided immediate corrective feedback in the posttest of intervention application to capitalize on the “teachable moment.” This feedback differed by dimension (Figure 2). For knowledge and PBC, a perfect score triggered an audio cue (“great”) and unlocked the next level. An imperfect score prompted “think again,” followed by display of the correct answers. For attitude and behavioral intention, a score ≥4 (on a 5-point scale) elicited positive audio feedback (“good choice”); a lower score triggered “think again,” followed by a review of key intervention content. No feedback was provided during the psychometric validation, methodological comparison, and baseline pretest of intervention application to ensure measurement purity. Of note, the feedback mechanism did not influence scoring, as scores were calculated prior to feedback delivery. The workflow of this gamified assessment and feedback system is illustrated in Figure 3.

**Figure 3:**
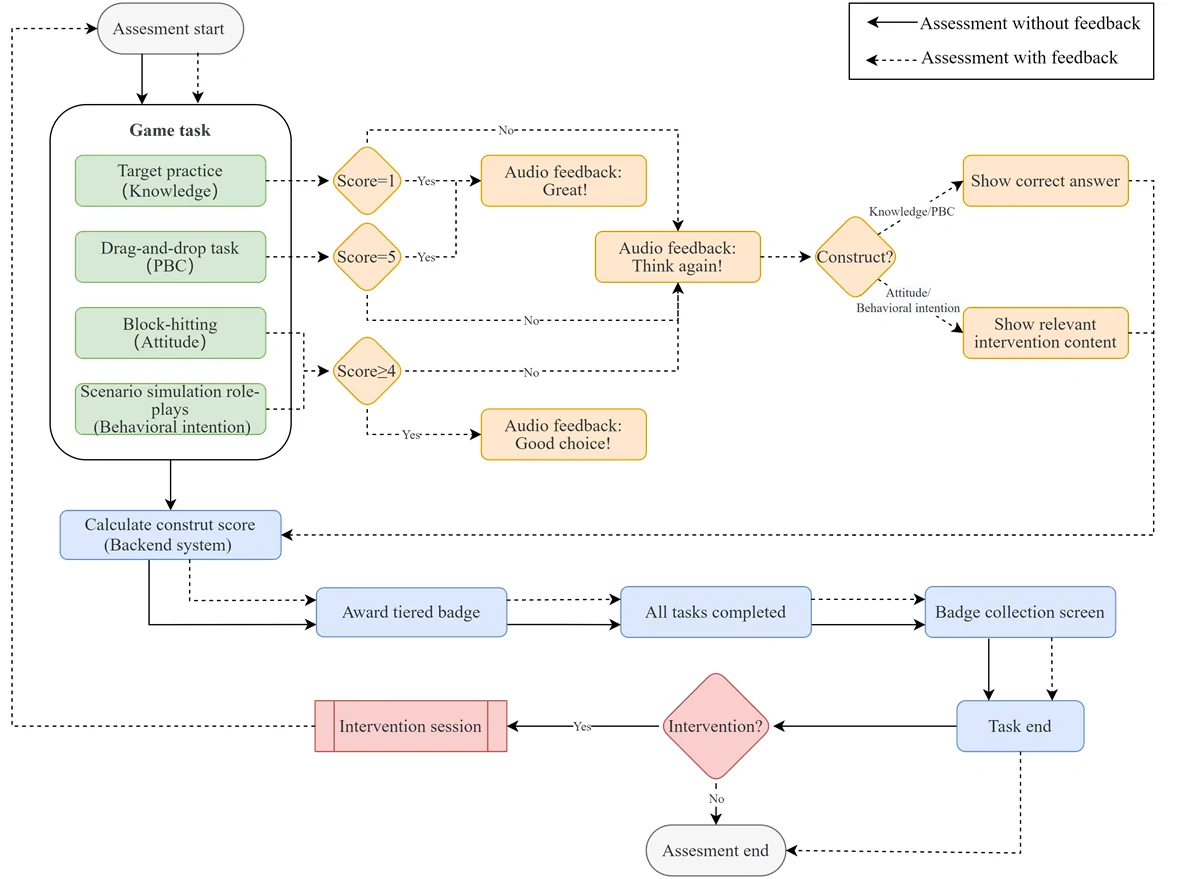
Operational workflow of the gamified assessment and feedback system, including game tasks, real-time audio feedback, badge awarding, and final badge collection (all phases, grades 4-6, Nanjing, China, 2025). Participants progress through 4 game tasks corresponding to the assessed dimensions (knowledge, attitude, perceived behavioral control (PBC), and behavioral intention). Solid arrows indicate the flow without feedback (phase 1, phase 2, and pretest of phase 3); dashed arrows indicate the flow with immediate corrective feedback, which was provided only during the posttest of phase 3: positive feedback for correct or high-scoring responses and corrective prompts, correct answers (knowledge and PBC) or relevant intervention content (attitude and behavioral intention) for incorrect or low-scoring responses. Upon task completion, the construct score is calculated in the backend, and a tiered badge is awarded. The final screen displays all earned badges before the assessment concludes. After all tasks are finished, participants either proceed to the intervention session (pretest of phase 3) or end the assessment (phase 1, phase 2, and posttest of phase 3).

Data quality was operationalized as the overall validity of each completed assessment, coded as a binary outcome (valid=1, invalid=0) based on predefined criteria.

In the methodological comparison study, participant experience (enjoyment and preference) was evaluated through self-reported ratings via a 5-point Likert scale.

In the longitudinal pre-post intervention study, the independent variable was time, reflecting the measurement occasion relative to the intervention (pretest vs posttest). The dependent variables were the scores of the 4 dimensions from the gamified tool. Covariates included in the analyses were class and gender.

### Data Collection

Data were collected through 2 primary modes: the gamified assessment automatically recorded responses on the devices, while the paper-based questionnaire was completed manually.

Data for phase 1 were collected at baseline and again at a 1-month follow-up. Data for phase 2 were collected using both the gamified and paper-based formats according to participants’ assigned sequences. Participant experience measures were collected immediately after this first assessment. Data for phase 3 were collected immediately before and after the intervention. Participants completed a 10-minute baseline pretest (T0), followed by the intervention session, and then a 10-minute posttest (T1) with the immediate feedback mechanism.

Initial data entry and basic cleaning were performed in Microsoft Excel 2024. At this stage, all negatively worded items were reverse-coded to ensure uniform scoring direction across the tool.

### Quality of Measurements

No formal training or reliability assessment was conducted for data collectors, as the gamified tool was fully automated and the paper-based questionnaire was administered following a standardized protocol. Repeated observations were not used.

### Masking

No masking was performed because the gamified and paper-based formats were visually distinct and the intervention was delivered in an open classroom setting.

### Psychometrics

A systematic 2-round expert review was conducted to establish the content validity of the gamified tool, ensuring relevance, clarity, and age-appropriateness for elementary school students. An initial review by 5 senior pharmacists (2 chief pharmacists and 3 associate chief pharmacists; all holding master’s degrees, with >10 years of experience, and representing pharmacy administration, clinical pharmacy, and outpatient pharmacy services within a tertiary children’s hospital) focused on medical accuracy and relevance, ensuring item clarity for subsequent reviewers. A subsequent review by 5 experienced elementary school teachers (>10 years of teaching) then evaluated child-appropriateness, with a focus on the comprehensibility of language and the realism of scenarios for the target age group. In both rounds, experts rated each item on a 4-point Likert scale (1=irrelevant, unclear, or inappropriate; 4=highly relevant, very clear, or very appropriate). The item-level content validity index (I-CVI) and the scale-level content validity index/average (S-CVI/Ave) were calculated. Given that each panel comprised 5 experts, an I-CVI of 1.00 (unanimous agreement) was required for item retention, resulting in the overall S-CVI/Ave also having to reach the maximum value of 1.00.

We examined the internal structure and measurement properties of the gamified tool by assessing construct validity using Rasch analysis. Item fit was assessed by examining the weighted (infit) and unweighted (outfit) mean-square (MNSQ) statistics for each item, with values between 0.5 and 1.5 considered indicative of acceptable model conformity. The essential unidimensionality of each scale was tested through a principal component analysis of the standardized residuals; support for unidimensionality required the eigenvalue of the first residual component to be below 3.0, coupled with a ratio of the second to first eigenvalue of less than 0.5. The discrimination power of each item was assessed via its point-measure correlation, where a value greater than 0.30 was taken to confirm the item’s positive alignment with the intended latent construct. Finally, the precision and reliability of each dimension’s measure were evaluated using the person separation reliability coefficient. A coefficient exceeding 0.70 was deemed acceptable, indicating that the tool possessed adequate power to distinguish between participants of different ability levels within the sample.

To evaluate test-retest reliability, we quantified the degree of absolute agreement between the 2 time points for each dimension by calculating the intraclass correlation coefficient (ICC). An ICC value of ≥0.70 was considered the threshold for acceptable temporal stability. Then, we complemented this by examining systematic score drift through 2-tailed paired *t* tests for each dimension. A nonsignificant result (*P*>.05) would indicate no meaningful average change in scores over the 1-month period, further supporting the stability of the measurement.

### Data Diagnostics

To ensure data integrity, clear validity criteria were applied to all collected questionnaires. For the paper-based questionnaire, manual completeness checks were performed, and any questionnaire with blank items was excluded. For the gamified assessment, the design enforced completion of each item before proceeding, eliminating data missingness at the item level. All responses from both formats were screened for patterned or nondifferentiated responding, which also led to exclusion. For pre-post comparisons, only participants with a complete pair of responses were considered.

Missing data were handled using complete-case analysis (listwise deletion). We also evaluated individual person-response validity by flagging participants whose infit or outfit statistics fell outside the 0.5 to 1.5 range, suggesting potential aberrant response patterns. When the estimated class-level variance was 0 (resulting in a singular fit), the ICC was set to 0.00. Model diagnostics were examined for linear models.

No additional statistical outliers were defined or excluded beyond the prespecified validity criteria. No formal distributional tests were performed as the planned analyses (GLMMs) are robust to moderate deviations from normality; residual diagnostics were examined for linear models. No additional data transformations were applied beyond those described in the Data Collection section.

### Analytic Strategy

All statistical analyses were conducted using R software (version 4.5.2; R Core Team) with packages *dplyr* (version 1.1.4), *tidyr* (version 1.3.1), and *ggplot2* (version 4.0.1) for data cleaning, transformation, and visualization, and dedicated packages for specific analyses as detailed below. All statistical tests were 2-sided with a significance level of α=.05.

In phase 1, construct validity analyses were conducted on the baseline data within the Rasch measurement framework. Given the different response formats across dimensions, we applied the dichotomous Rasch model to the binary-scored knowledge items and the partial credit model to the polytomous items measuring attitude, PBC, and behavioral intention. All models were estimated via the marginal maximum likelihood method, using robust convergence criteria (maximum iterations=1000, increment factor=1.05) using the *TAM* package (version 4.3-25), complemented by the *psych* package (version 2.5.6). Regarding item performance, we calibrated item difficulty/location parameters and their standard errors. Test-retest reliability was quantified by comparing data from the 2 time points using a 2-way mixed-effects model for absolute agreement (ICC 3-1) via the *psych* package (version 2.5.6). Paired *t* tests (base R) were used to examine systematic score drift over the 1-month interval.

In phase 2, measurement equivalence was evaluated through a multimethod approach using data from both assessment sessions. To ensure the validity of pooling data from the crossover design, we tested for order effects using a 2 × 2 mixed-design ANOVA (base R), with sequence (A or B) as the between-subjects factor and modality (gamified or paper) as the within-subjects factor. A nonsignificant sequence × modality interaction (*P*>.05) was taken to indicate the absence of a meaningful order effect, permitting data pooling. With order effects assessed, absolute agreement between the 2 modalities was quantified using ICC(3,1). We used paired *t* tests to examine systematic mean differences, with Cohen *d* (*effsize* package, version 0.8.1) quantifying the effect size. Concurrently, Bland-Altman analysis (*BlandAltmanLeh* package, version 0.3.1) was applied to visualize individual-level differences, estimate mean bias, and calculate 95% limits of agreement. Finally, to assess the consistency of categorical decisions made from the 2 tools, scores for each dimension were dichotomized into “high” and “low” groups based on the median. We then computed both Cohen κ coefficient (*irr* package, version 0.84.1) and simple percentage agreement to evaluate the classification consistency between modalities. To ensure a fair comparison of initial exposure and minimize carryover effects in the crossover design, the superiority analysis used only the data from the first assessment session (gamified assessment from sequence A; paper-based assessment from sequence B). We compared the proportion of valid responses between the 2 modalities using a chi-square test (base R) and calculated the validity rate with its 95% CI for each. Participant experience was compared using independent-samples *t* tests (base R) with Cohen *d* as the effect size.

In phase 3, baseline comparability was assessed using only the pretest data. We used 1-way ANOVA (base R) to compare class-level baseline scores for all 4 dimensions: knowledge (percentage correct, 0%-100%), attitude, PBC, and behavioral intention (each a Likert sum score ranging from 4 to 20). To quantify between-class clustering at baseline, we estimated the ICC(1) for each dimension using a 1-way random-effects model with a random intercept for class (*lme4* package, version 1.1-38) and extracted the ICC (*performance* package, version 0.15.3). Responsiveness was evaluated using GLMMs on paired pre-post test data using the *lme4* (version 1.1-38), *lmerTest* (version 3.2-0), and *emmeans* (version 2.0.1) packages. Considering the distinct measurement scales, different model families were specified: a Poisson mixed model with a log link for the count-type knowledge score, and linear mixed models for the continuous attitude, PBC, and behavioral intention scores. Each model included time (T0/T1), class, gender, and the class × time interaction as fixed effects, and incorporated a random intercept per participant to account for repeated measurements. The class × time interaction was tested using likelihood-ratio tests (Poisson model) or *F* tests (linear models). If the interaction was significant, simple-effects analyses with Tukey-adjusted *P* values were performed to clarify differential change across classes. To quantify the magnitude of within-class change, the effect size was estimated as Cohen *d* for paired data, derived from the model-estimated marginal means and pooled SD.

## Results

### Sample Characteristics

The flowchart of participant enrollment is detailed in Figure 4. In phase 1, the full sample (n=81) provided data for the assessment of construct validity. For test-retest reliability analysis, 77 of the original 81 participants (39/77, 50.6% female) completed both assessments. Four participants were excluded due to nonresponse at the second time point (1-month follow-up), resulting in 4 missing data points (4/81, 4.9%). In phase 2, all 85 participants provided data for the analysis of methodological superiority, which used first-assessment data only. For the sequence effects and equivalence analyses, after confirming no significant order effect, data from participants with valid responses at both time points were pooled across sequences, yielding a final analytic sample of 76 paired observations (39/76, 51.3% female). Nine participants were excluded because at least 1 of their 2 assessments was invalid (9/85, 10.6%). In phase 3, the entire sample with complete preintervention and postintervention data was used in the longitudinal GLMM analysis to assess the tool’s responsiveness. Detailed sample sizes for each analysis are presented in Table 1.

**Figure 4:**
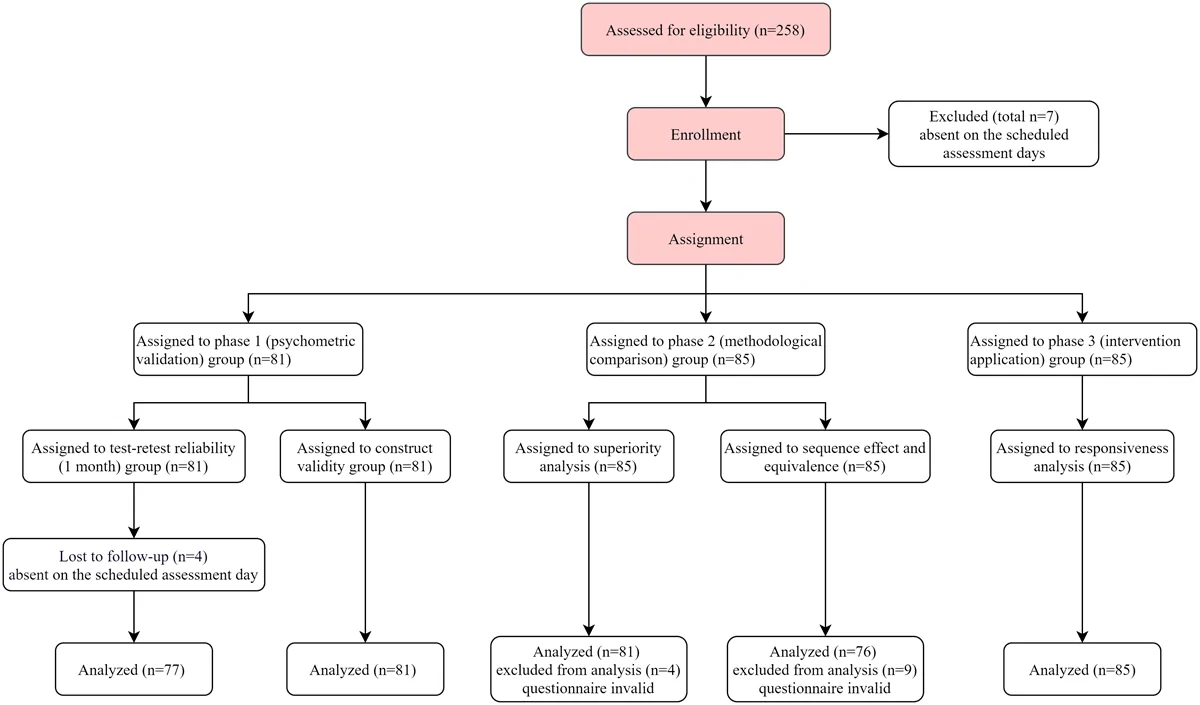
Participant flow diagram for the 3 study phases (psychometric validation, methodological comparison, and intervention application) among elementary school children (grades 4-6) in Nanjing, China (2025). Numbers indicate participants assessed for eligibility (n=258), excluded due to absence (n=7), allocated to each phase (phase 1: n=81, phase 2: n=85, and phase 3: n=85), and subsequently included in the analyses. Attrition (loss to follow-up, n=4 in test-retest reliability in phase 1) and exclusions from analysis (superiority analysis in phase 2: n=4 due to invalid questionnaires; sequence effect and equivalence analysis in phase 2: n=9 due to invalid questionnaires) are explicitly indicated.

**Table 1:** Sample characteristics and analytic sample sizes by study phase for elementary school children (grades 4-6) in Nanjing, China (2025).

Phase (classes; grades) and analysis		Female, n/N (%)
Psychometric validation (2; 4 and 6)		40/81 (49.4)
	Construct validity	40/81 (49.4)
	Test-retest reliability	39/77 (50.6)
Methodological comparison (2; 5 and 6)		42/85 (49.4)
	Sequence effect	39/76 (51.3)
	Equivalence	39/76 (51.3)
	Superiority	42/85 (49.4)
Intervention application (2; 5 and 6)		41/85 (48.2)
	Responsiveness	41/85 (48.2)

### Phase 1: Psychometric Validation

#### Content Validity

In the first-round expert review (pharmacy experts), 15 of 18 initial items directly achieved an I-CVI of 1.00. One item was deleted due to low relevance (I-CVI=0.20), and 2 items were revised based on clarity feedback and subsequently reached consensus (I-CVI=1.00). In the second round (teacher experts), 11 of the 17 revised items directly achieved an I-CVI of 1.00 for age-appropriateness; the remaining 6 items were modified per expert suggestions and then also achieved unanimous approval. Consequently, all retained items attained an I-CVI of 1.00, resulting in an S-CVI/Ave of 1.00. The content validity indices are summarized in Table 2. These results indicate fine content relevance, clarity, and age-appropriateness of the gamified assessment tool.

**Table 2:** Psychometric properties (content validity, item fit, unidimensionality, person separation reliability, and 1-month test-retest reliability) of the gamified medication literacy tool in a validation sample (phase 1, n=81, grades 4 and 6, Nanjing, China, 2025).

Domain	Content validity (I-CVI^a^); item fit (mean infit MNSQ^b^)	Unidimensionality^c^ (residual eigenvalue/ratio)	Person separation reliability^d^	Test-retest reliability, ICC^e^ (95% CI)
Knowledge	1.00; 1.01	2.25/0.40	0.69	0.76 (0.64-0.84)
Attitude	1.00; 1.07	2.14/0.34	0.72	0.92 (0.88-0.95)
Perceived behavioral control	1.00; 1.04	2.19/0.34	0.71	0.86 (0.79-0.91)
Behavioral Intention	1.00; 1.04	2.21/0.38	0.74	0.86 (0.79-0.91)

^a^I-CVI: item-level content validity index (unanimous agreement among 5 experts per panel; I-CVI=1; scale-level content validity index/average [S-CVI/Ave]=1.00).

^b^MNSQ: mean-square statistic (item fit was considered acceptable if infit MNSQ was between 0.5 and 1.5).

^c^Unidimensionality criteria: residual eigenvalue (first residual component eigenvalue) <3.0 and ratio of the second to first eigenvalue <0.5.

^d^Person separation reliability ≥0.70.

^e^ICC: intraclass correlation coefficient. ICC(3,1) was calculated over a 1-month interval using a 2-way mixed-effects model with absolute agreement; ICC≥0.70 was considered acceptable).

#### Construct Validity

Mean infit MNSQ values were between 1.01 and 1.07, and mean outfit MNSQ values were between 0.83 and 1.23. Individual infit and outfit MNSQ values for all 17 items were predominantly within 0.5 to 1.5. All item-level and person-level values were within the acceptable range, indicating adequate model-data fit and no aberrant response patterns. The eigenvalue of the first residual component ranged from 2.14 to 2.25 (all <3.0), and the unidimensionality ratio ranged from 0.34 to 0.40 (all <0.5), supporting the essential unidimensionality of each dimension. Item difficulty parameters spanned from –2.07 to 1.59 logits for knowledge, from –0.81 to 0.44 for attitude, from 0.40 to 0.87 for PBC, and from –0.66 to 0.27 for behavioral intention. Point-measure correlations ranged from 0.67 to 0.73, exceeding the 0.30 criterion and confirming satisfactory item discrimination. Person separation reliability ranged from 0.69 to 0.74, approaching or exceeding the acceptable threshold of 0.70 for group-level comparisons. The Rasch-based model fit statistics, unidimensionality indices, and reliability estimates for each domain are presented in Table 2. Collectively, these findings support the structural validity of the gamified assessment tool.

#### Test-Retest Reliability

Across the 4 dimensions, ICC(3,1) values ranged from 0.76 to 0.92, all exceeding the prespecified criterion of 0.70 and indicating good to excellent temporal stability. The narrow 95% CIs indicate precise estimates of temporal stability (Table 2). No statistically significant mean differences were observed between time point 1 and time point 2 (knowledge: *P*=.41; attitude: *P*=.09; PBC: *P*=.39; behavioral intention: *P*=.71), suggesting no systematic score drift over the 1-month interval.

### Phase 2: Methodological Comparison

#### Equivalence Analysis

The 2 × 2 mixed-design ANOVA found no significant sequence (A or B) × modality (gamified or paper) interaction for any dimension (all *P*>.05, detailed in Table 3), indicating the absence of a meaningful order effect. This finding also confirms that the 10-minute washout period was sufficient to avoid systematic recall bias. Therefore, data from both administration sequences were pooled for subsequent equivalence analyses. The results of the multimethod equivalence analysis are presented in Table 3. For all 4 dimensions, ICC(3,1) values ranged from 0.94 to 0.99, with narrow 95% CIs indicating precise estimates. Paired *t* tests revealed no statistically significant mean score differences (all *P*>.05), with associated Cohen *d* values from –0.017 to 0.047 and narrow 95% CIs indicating high precision. Bland-Altman analyses showed mean bias values close to 0, with 95% limits of agreement ranging from ±0.16 to ±0.63 logit; the 95% CIs for the mean biases were also narrow, indicating high precision. These negligible biases and narrow limits of agreement are visually summarized in the Bland-Altman plots presented in Figure 5. Cohen κ coefficients ranged from 0.87 to 0.97, and percentage agreement from 93.4% to 98.7%. These convergent findings across multiple indices support the measurement equivalence of the 2 modalities, indicating that the gamified tool produces scores comparable to the paper-based format.

**Figure 5:**
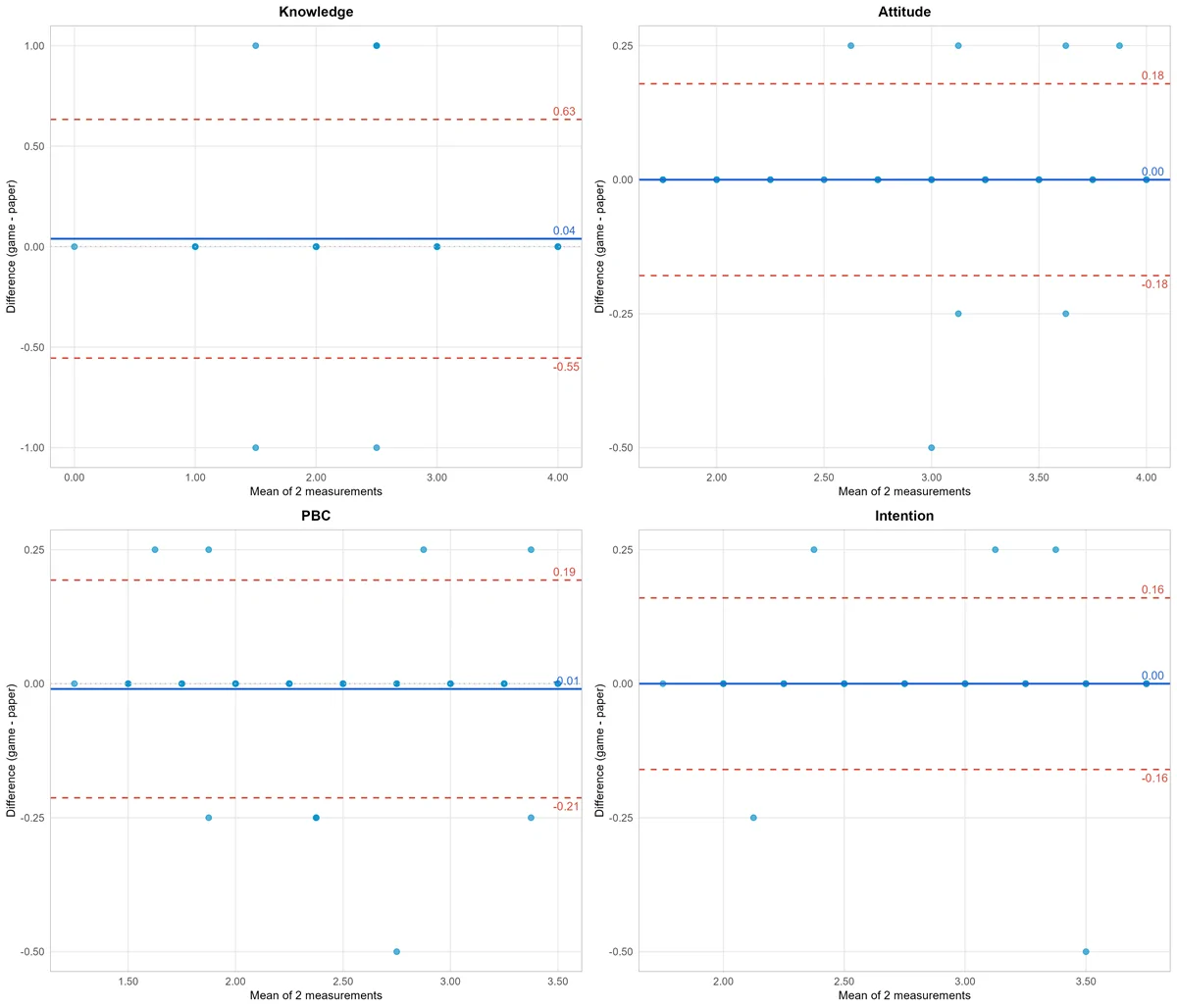
Bland-Altman plots comparing gamified vs paper-based medication literacy scores for each domain in a randomized crossover design (phase 2, n=76 paired observations, grades 5 and 6, Nanjing, China, 2025). The solid horizontal line represents the mean bias (average difference between modalities); the dashed lines represent the 95% limits of agreement (mean bias SD 1.96). PBC: perceived behavioral control.

**Table 3:** Measurement equivalence between gamified and paper-based medication literacy assessments using a randomized crossover design (phase 2, n=85, grades 5 and 6, Nanjing, China, 2025). Order effect was tested using a 2 × 2 mixed-design ANOVA (sequence × modality interaction).

Domain	Order effect, *F* test (*df*); *P* value	ICC^a^ (95% CI)	Score difference^b^, *P* value; Cohen *d* (95% CI)	Bland-Altman bias (95% LoA^c^; 95% CI)	Decision consistency, κ (agreement %)
Knowledge	0.38 (1, 66); .54	0.94 (0.90 to 0.96)	.26; 0.047 (–0.04 to 0.13)	0.039 (–0.554 to 0.633; 0.19 to 0.27)	0.87 (93.4)
Attitude	0.01 (1, 66); .94	0.99 (0.98 to 0.99)	>.99; 0.000 (–0.04 to 0.04)	0.000 (–0.179 to 0.179; 0.06 to 0.06)	0.92 (96.1)
Perceived behavioral control	0.01 (1, 66); .93	0.98 (0.97 to 0.99)	.41; –0.017 (–0.06 to 0.02)	–0.010 (–0.213 to 0.193; 0.10 to 0.08)	0.92 (96.1)
Behavioral intention	0.00 (1, 66); .97	0.99 (0.98 to 0.99)	>.99; 0.000 (–0.03 to 0.03)	0.000 (–0.160 to 0.160; 0.06 to 0.06)	0.97 (98.7)

^a^ICC: intraclass correlation coefficient; ICC(3,1) was calculated using a 2‑way mixed‑effects model with absolute agreement.

^b^Score differences were tested using a paired *t* test; Cohen *d* indicates the effect size.

^c^LoA: limits of agreement.

#### Superiority Analysis

The results of the superiority analysis are summarized in Table 4. Figure 6 provides an integrated overview of the comparisons in terms of experience (enjoyment and preference). Overall tool validity did not differ significantly between modes (*χ*^2^_1_=2.3; *P*=.13), with rates of 100% (42/42) for the gamified and 90.7% (39/43) for the paper-based assessment. The wide overlap of these 95% CIs is consistent with the nonsignificant chi-square test. In contrast, the gamified tool yielded substantially higher scores for both enjoyment and preference, with all corresponding *t* tests being highly significant (*P*<.001). The narrow 95% CIs confirm the precision of these estimates. These results demonstrate that while the gamified tool achieves measurement parity, it offers a clear advantage in user experience.

**Table 4:** Methodological superiority of gamified vs paper-based medication literacy assessment: data quality and participant experience (enjoyment and preference) in the first assessment of a crossover study (phase 2, n=85, grades 5 and 6, Nanjing, China, 2025).

Comparison metric			Gamified tool		Paper-based questionnaire		Test statistic, chi-square (*df*) or *t* test (*df*)		*P* value		Cohen *d* (95% CI)
Data quality^a^											
	Valid response rate, n/N (%); 95% CI	42/42 (100); 91.6%-100%		39/43 (90.7); 77.4%-96.9%		2.29 (1)^b^		.13		—^c^	
Participant experience^d^											
	Enjoyment, mean (SD)	4.26 (0.94)		2.46 (0.85)		9.01 (79)^e^		<.001		2.00 (1.46- 2.55)	
	Preference, mean (SD)	4.29 (0.83)		2.82 (0.97)		7.30 (79)^e^		<.001		1.62 (1.11- 2.13)	

^a^Data quality was assessed based on predefined completion criteria.

^b^Indicates a chi-square statistic.

^c^Not applicable.

^d^Participant experience was rated on a 5-point Likert scale immediately after the first assessment. Independent-samples *t* tests were used to compare experience between groups.

^e^Indicates a *t* test statistic.

**Figure 6:**
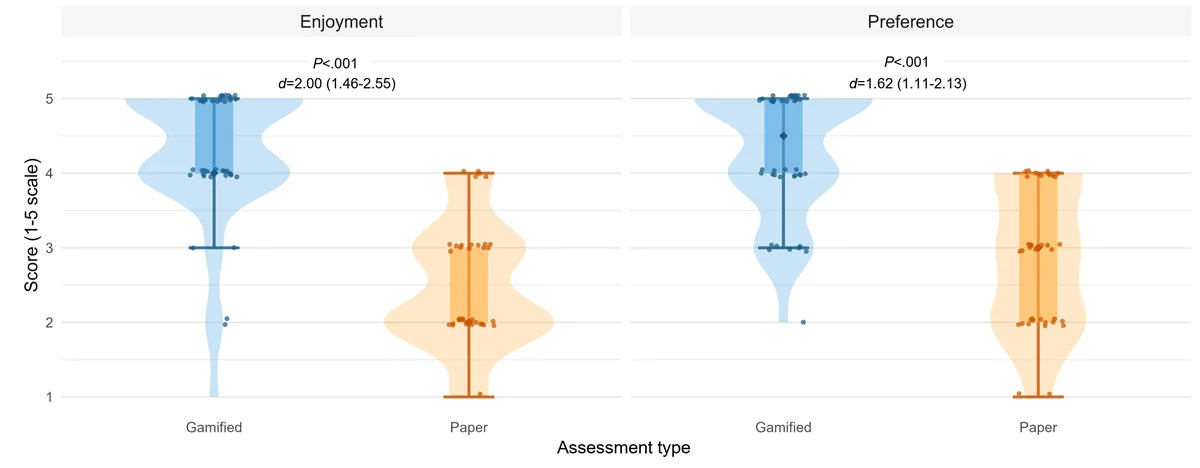
Distribution of enjoyment and preference scores by assessment format (violin plot) among elementary school children (grades 5 and 6) in Nanjing, China (2025). Mean scores for enjoyment and preference. Error bars represent 95% CIs.

### Phase 3: Intervention Application

#### Descriptive Statistics

The intervention cohort was from 2 intact classes (A and B), each representing a distinct grade level (grades 6 and 5, respectively). Class-level comparisons revealed a significant baseline difference for knowledge (*P*=.007), with class A (grade 6) scoring higher than class B (grade 5). In contrast, no significant between-class differences were observed at baseline for attitude (*P*=.65), PBC (*P*=.43), or behavioral intention (*P*=.49). ICC(1) for knowledge was 0.14 (95% CI 0.00-0.53), indicating low clustering. For attitude, PBC, and behavioral intention, the ICC(1) estimates were 0.00 (95% CI 0.00-0.18, 95% CI 0.00-0.14, and 95% CI 0.00-0.16, respectively), indicating negligible clustering.

#### Responsiveness Analysis

The GLMM results are presented in Table 5. All 95% CIs for the time effects (*β* and incidence rate ratio [IRR]) exclude the null value, indicating precise and reliable estimates of pre-post change. The Poisson mixed model for knowledge also showed a significant improvement (*P*<.001). For the continuous outcomes (attitude, PBC, and behavioral intention), linear mixed models revealed significant main effects of time (all *P*<.001). The time × class interaction effects were nonsignificant across all dimensions (all *P*≥.19), indicating consistent pre-post changes across classes.

**Table 5:** GLMM^a^ results for intervention responsiveness of the gamified medication literacy tool in a pre-post design (phase 3, n=85, grades 5 and 6, Nanjing, China, 2025).

Domain (model) and fixed effect		*β*^b^ (95% CI) or IRR^c^ (95% CI)	*z* score, chi-square (*df*), *t* test (*df*), or *F* test (*df*)	*P* value
Knowledge (Poisson)				
	Time (posttest vs pretest)	1.52 (1.22-1.89)	3.72^d^	<.001
	Time × class	—	0.38 (1)^e^	.54
Attitude (linear)				
	Time (posttest vs pretest)	1.74 (1.25-2.22)	7.04 (83)^f^	<.001
	Time × class	—	0.00 (1, 83)^g^	.99
Perceived behavioral control (linear)				
	Time (posttest vs pretest)	2.86 (2.18-3.53)	8.30 (83)^f^	<.001
	Time × Class	—	0.03 (1, 83)^g^	.85
Behavioral intention (linear)				
	Time (posttest vs pretest)	2.33 (1.51-3.15)	5.59 (83)^f^	<.001
	Time × class	—	1.74 (1, 83)^g^	.19

^a^GLMM: generalized linear mixed model.

^b^Unstandardized coefficient (linear models).

^c^IRR: incidence rate ratio (Poisson model). All models included random intercepts for class and individual (ID). For the count outcome (knowledge), the interaction *P* value was obtained from a likelihood ratio test (chi-square test); for continuous outcomes, the time × class interaction was tested using an *F* test.

^d^Indicates a *z* score statistic.

^e^Indicates a chi-square statistic.

^f^Indicates a *t* test statistic.

^g^Indicates an *F* test statistic.

The within-class effect sizes (Cohen *d*; Figure 7) with 95% CIs were as follows: for knowledge, class A was 1.53 (95% CI 1.08-1.97) and class B was 1.46 (95% CI 1.02-1.88); for attitude, both classes were 1.09 (95% CI 0.70-1.47 for class A, 95% CI 0.70-1.46 for class B); for PBC, class A was 1.29 (95% CI 0.87-1.70) and class B was 1.23 (95% CI 0.83-1.63); and for behavioral intention, class A was 0.75 (95% CI 0.40-1.09) and class B was 0.70 (95% CI 0.36-1.03). All 95% CIs were relatively narrow and did not include 0, indicating precise and statistically significant changes from pretest to posttest. Figure 7 presents these effect sizes graphically.

**Figure 7:**
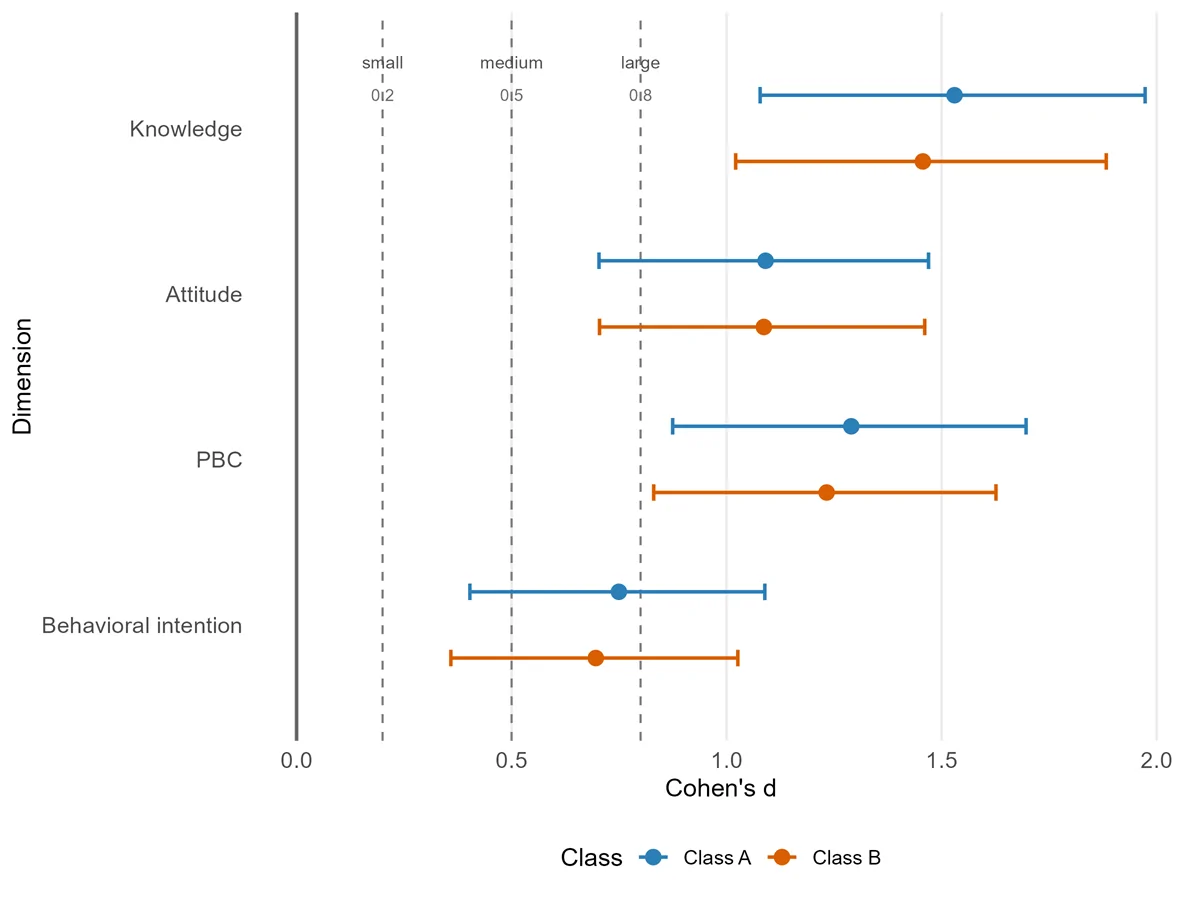
Forest plot of within-class effect sizes (Cohen d) for the 4 medication literacy dimensions (knowledge, attitude, perceived behavioral control [PBC], and behavioral intention) by class (class A and class B) in a pre-post intervention study (phase 3, n=85, grades 5 and 6, Nanjing, China, 2025). Cohen d was calculated using paired t tests (preintervention vs postintervention). Each solid point represents the point estimate for one class in a given dimension; error bars show the 95% CI. The vertical dashed lines at d=0.2, d=0.5, and d=0.8 indicate small, medium, and large effect size benchmarks, respectively.

## Discussion

### Principal Results

The primary objective of this study was to develop and validate a gamified assessment tool for measuring medication literacy in elementary school children, to compare it with a traditional paper-based format, and to evaluate its responsiveness to an educational intervention. The results across the 3 phases collectively show that the tool has adequate psychometric properties, is measurement-equivalent to the paper-based questionnaire, is preferred by children, and can detect changes associated with the intervention consistently across classrooms. The psychometric validation results indicate that the newly developed gamified assessment tool demonstrates adequate psychometric properties for research use with elementary school students. The perfect content validity indices confirm that the gamified content is not only theoretically sound but also contextually appropriate and comprehensible for elementary school students [38]. Rasch analysis supported the structural validity of the 4 hypothesized constructs within the gamified format. The essential unidimensionality and acceptable item fit of all dimensions indicate that children’s interactions with the game-like interface produced response data consistent with the underlying measurement models [39,40]. The point-measure correlations exceeding the criterion further verify that each item effectively discriminated along its intended latent trait [40]. The person separation reliability coefficients of attitude, PBC, and behavioral intention were in the acceptable range for group-level analysis [40]. The slightly lower value for knowledge is not surprising, given that binary items typically yield lower person separation reliability than polytomous items with a comparable number of items [41]. Overall, the tool provides reasonable precision for distinguishing between cohorts with different ability levels across all dimensions. These Rasch-based findings are consistent with the methodological approach used in the validation of other child-focused assessment tools [42], supporting the appropriateness of this framework for pediatric samples. The test-retest results showed strong temporal stability, indicating that the engaging format did not introduce response instability or significant practice effects upon readministration [43]. In summary, phase 1 establishes that the methodological innovation of gamification can yield measurements that meet rigorous psychometric standards, providing a reliable and valid means to assess the target constructs. This lays the foundation for its subsequent comparison against traditional methods in phase 2 and its application in evaluating intervention effects in phase 3.

Turning to the methodological comparison, the results indicate that the 2 modalities produce effectively equivalent measurements, while the gamified version addresses a key limitation of traditional paper-based questionnaires by significantly enhancing participant engagement. The equivalence analysis confirmed that the 2 formats measure the same constructs [44]. After establishing the absence of order effects, pooled data revealed high ICCs across all dimensions [45]. This was consistent with other metrics: no systematic mean differences were detected, Bland-Altman plots showed minimal bias [46], and decision consistency was high [47]. These convergent results across multiple indices support measurement equivalence, satisfying a fundamental prerequisite for considering the gamified tool a valid alternative to the paper-based format [44]. Where the tools meaningfully diverge is in the participant experience—the core value proposition of this methodological innovation. While both yielded data of comparable quality—reflected in the high and nonsignificantly different validity rates—this surface-level equivalence in completion likely reflects the strong subjective norms of the school setting [32], which can ensure task compliance even for less engaging formats. The gamified tool, in contrast, was rated substantially higher in both enjoyment and preference, consistent with previous reports that gamified assessments are more engaging for children [27]. This shift proactively addresses a core challenge to the validity of child self-report data [23]. It moves beyond reliance on external compliance by transforming the assessment into a more motivationally compatible activity—a transformation that fosters greater willingness to engage and reduces inattentive responding [48]. In conclusion, this comparative phase demonstrates that the gamified tool successfully bridges a common gap in assessment methodology. It achieves measurement equivalence with the paper-based format while simultaneously enhancing the engagement necessary for high-quality data collection from children. This achievement validates it as a methodologically robust instrument appropriate for the target population, well-suited for application in the subsequent intervention study.

In terms of intervention responsiveness, the gamified assessment tool demonstrated satisfactory responsiveness [49], detecting significant changes across all 4 medication literacy domains following a targeted educational intervention. The moderate to large effect sizes indicated that the tool captured substantial changes across the 4 domains [50]. Notably, the pre-post changes were consistent across classrooms for all dimensions, despite a significant baseline difference in knowledge scores. This finding carries practical importance: students with lower baseline knowledge showed comparable improvements to those with higher baseline scores. This pattern aligns with recent evidence that game-based interventions can improve outcomes equitably regardless of baseline disparities [51]. More broadly, school-based health literacy interventions have been recognized as a promising strategy for reducing health inequalities [52]. The tool’s ability to detect uniform changes across classrooms may be useful for evaluating health education initiatives in diverse settings [53,54]. Taken together, these results confirm that the tool is capable of capturing meaningful pre-post changes in children’s medication literacy, supporting its utility as an outcome measure for school-based health promotion initiatives.

### Limitations

Several limitations should be acknowledged. The intervention used a single-group pre-post design without a control condition. While the primary aim was to evaluate the tool’s responsiveness rather than intervention efficacy, the absence of a comparison group limits causal inference regarding the observed changes. Therefore, our results should be interpreted as evidence that the tool can detect changes associated with the intervention, not as proof that the intervention caused those changes. Moreover, the immediate posttest administration may have introduced short-term recall effects, potentially overestimating immediate improvements relative to long-term retention. This study did not examine whether the observed changes were sustained over time, leaving the question of their long-term sustainability unanswered. Future studies should consider incorporating a control arm and longer-term follow-up assessments to strengthen causal conclusions and evaluate the durability of the measured changes.

All 3 phases recruited independent samples from a single elementary school—a decision driven by practical considerations of conducting research in real-world school settings, though it may constrain generalizability to other educational environments or age groups. Replicating these findings across multiple schools with diverse demographics would help establish the tool’s broader applicability. The sample sizes are acceptable but modest; the Rasch sample approaches the lower bound for stable calibration, and the small number of classes (2 per phase) limits the precision of random-effect estimates. While the absence of significant order effects indicates that the 10-minute washout period was adequate for this study, longer intervals (eg, 24 hours) could be considered in future crossover designs to further eliminate any potential recall of responses. Although the missing data proportion was low (4/81, 4.9% in phase 1, none in phase 3) and the exclusions in phase 2 (9/85, 10.6%) were based on questionnaire validity, complete-case analysis may introduce some bias. We allocated 10 minutes for each assessment session based on pilot estimates, but individual completion times were not systematically recorded. Future studies should collect such feasibility data to better inform practical implementation in school settings.

### Conclusions

We conclude that a gamified assessment tool can be both psychometrically rigorous, engaging for elementary school children, and responsive to detecting intervention-associated changes. This study demonstrates the use of a fully gamified format to target children’s medication literacy, directly compares it against a paper-based questionnaire using a randomized crossover design, and shows consistent pre-post changes across classrooms with different baseline knowledge levels. Unlike many existing child health measures that rely on adult proxy reports or adapted adult scales, our tool is child-centered, directly validated with students, and designed to enhance engagement. Beyond providing an effective alternative to traditional paper-based questionnaires, the tool may offer a practical solution for evaluating school-based medication literacy programs. The ability to detect uniform changes across classrooms points to its potential to support health equity. We suggest that as digital health interventions become more common in schools, validated gamified assessments like this one may provide practical outcome measures for evaluating their effects. Future research should extend this tool to other health domains and test its utility in longitudinal, multisite studies, ideally with larger samples (eg, ≥150 participants) and more clusters (eg, ≥4 schools) to improve generalizability.

## Data Availability

The datasets generated or analyzed during this study are available from the corresponding author on reasonable request.

## Appendix

Multimedia Appendix 1 English translations, item justifications, scoring rules, badge designs, and rationale for the gamified assessment tool.

## Abbreviations

CHERRIES: Checklist for Reporting Results of Internet E-Surveys
GLMM: generalized linear mixed model
ICC: intraclass correlation coefficient
I-CVI: item-level content validity index
IRR: incidence rate ratio
KAP: knowledge-attitude-practice
MNSQ: mean-square
PBC: perceived behavioral control
S-CVI/Ave: scale-level content validity index/average
TPB: theory of planned behavior

## References

[1] Cai T, Yu H, Yao Y, Xia Y, Zhou Z, Shen J, Lou J (2026). A scoping review of national policies for healthy China from 2008 to 2024. BMC Public Health.

[2] Ning C, Pei H, Huang Y, Li S, Shao Y (2024). Does the Healthy China 2030 policy improve people's health? Empirical evidence based on the difference-in-differences approach. Risk Manag Healthc Policy.

[3] Jiang Z, Jiang W (2021). Health education in the Healthy China Initiative 2019-2030. China CDC Wkly.

[4] Nutbeam D, Lloyd JE (2021). Understanding and responding to health literacy as a social determinant of health. Annu Rev Public Health.

[5] World Health Organization (2017). Shanghai declaration on promoting health in the 2030 Agenda for Sustainable Development. Health Promot Int.

[6] Jordão I, Paiva P, Dias P, António N, Parente F (2025). Health literacy and medication adherence in polypharmacy: a systematic review for clinical practice. Cureus.

[7] Duong H, Chang P (2024). Topics included in health literacy studies in Asia: a systematic review. Asia Pac J Public Health.

[8] Li Y, Lv X, Liang J, Dong H, Chen C (2022). The development and progress of health literacy in China. Front Public Health.

[9] Alqarni AS, Pasay-An E, Saguban R, Cabansag D, Gonzales F, Alkubati S, Villareal S, Lagura GAL, Alshammari SA, Aljarboa BE, Mostoles R (2023). Relationship between the health literacy and self-medication behavior of primary health care clientele in the Hail Region, Saudi Arabia: implications for public health. Eur J Investig Health Psychol Educ.

[10] Avazeh Y, Rezaei S, Bastani P, Mehralian G (2022). Health literacy and medication adherence in psoriasis patients: a survey in Iran. BMC Prim Care.

[11] Rezaei S, Vaezi F, Afzal G, Naderi N, Mehralian G (2022). Medication aadherence and health literacy in patients with heart failure: a cross-sectional survey in Iran. Health Lit Res Pract.

[12] Zaeh SE, Ramsey R, Bender B, Hommel K, Mosnaim G, Rand C (2022). The impact of adherence and health literacy on difficult-to-control asthma. J Allergy Clin Immunol Pract.

[13] Jia Q, Wang H, Wang L, Wang Y (2022). Association of health literacy with medication adherence mediated by cognitive function among the community-based elders with chronic disease in Beijing of China. Front Public Health.

[14] Guo S, Naccarella L, Riggs E (2023). Promoting child health equity through health literacy. Children (Basel).

[15] Chandrakar A, Ramasamy S, Galhotra A, Shenoy MS (2025). Maternal health literacy (MHL) for improved maternal and child outcomes: a scoping review. Indian J Community Med.

[16] Ahmadi F, Karamitanha F (2023). Health literacy and nutrition literacy among mother with preschool children: what factors are effective?. Prev Med Rep.

[17] Xu X, Wang Z, Li X, Li Y, Wang Y, Wu X, Hao L, Wang X (2022). Acceptance and needs of medication literacy education among children by their caregivers: a multicenter study in mainland China. Front Pharmacol.

[18] Zhang Y, Wang X, Cai J, Yang Y, Liu Y, Liao Y, Zhou Y, He B, Wen W, Zhuang Q, Lin Y (2022). Status and influencing factors of medication literacy among Chinese caregivers of discharged children with Kawasaki disease. Front Public Health.

[19] Shahar S, Shahar HK, Muthiah SG, Mani KKC (2022). Evaluating health education module on hand, food, and mouth diseases among preschoolers in Malacca, Malaysia. Front Public Health.

[20] McAdams E, Tingey B, Ose D (2022). Train the trainer: improving health education for children and adolescents in Eswatini. Afr Health Sci.

[21] Bakhtiarvand SZ, Rahaei Z, Sadeghian HA, Fatehi F, Soltani S, Zareiyan A, Nutbeam D (2025). The constructs of health literacy in children: a systematic review. BMC Public Health.

[22] Standage-Beier CS, Ziller SG, Bakhshi B, Parra OD, Mandarino LJ, Kohler LN, Coletta DK (2022). Tools to measure health literacy among adult hispanic populations with type 2 diabetes mellitus: a review of the literature. Int J Environ Res Public Health.

[23] Wang MD, Zang X (2025). Can we trust children’s self-reports? Examining socially desirable responses in elementary school surveys. Int J Educ Methodol.

[24] Van Boxtel W, Jerković-Ćosić K, Schoonmade LJ, Chinapaw MJM (2024). Health literacy in the context of child health promotion: a scoping review of conceptualizations and descriptions. BMC Public Health.

[25] Adhikari P, Paudel K, Bhusal S, Gautam K, Khanal P, Adhikari TB, Shrestha R, Shrestha SM (2024). Health literacy and its determinants among school-going children: a school-based cross-sectional study in Nepal. Health Promot Int.

[26] Sarhan MBA, Fujiya R, Kiriya J, Htay ZW, Nakajima K, Fuse R, Wakabayashi N, Jimba M (2023). Health literacy among adolescents and young adults in the Eastern Mediterranean region: a scoping review. BMJ Open.

[27] Garvey W, Schembri R, Oberklaid F, Hiscock H (2022). A health-education intervention to improve outcomes for children with emotional and behavioural difficulties: protocol for a pilot cluster randomised controlled trial. BMJ Open.

[28] Martínez-Miranda J, Espinosa-Curiel IE (2022). Serious games supporting the prevention and treatment of alcohol and drug consumption in youth: scoping review. JMIR Serious Games.

[29] Li M, Ma S, Shi Y (2023). Examining the effectiveness of gamification as a tool promoting teaching and learning in educational settings: a meta-analysis. Front Psychol.

[30] Wang M, Xu J, Zhou X, Li X, Zheng Y (2025). Effectiveness of gamification interventions to improve physical activity and sedentary behavior in children and adolescents: systematic review and meta-analysis. JMIR Serious Games.

[31] Gkintoni E, Vantaraki F, Skoulidi C, Anastassopoulos P, Vantarakis A (2024). Gamified health promotion in schools: the integration of neuropsychological aspects and CBT—a systematic review. Medicina (Kaunas).

[32] da Silva Pinho A, Slagter S, Gradassi A, Molleman L, Braams BR, van den Bos W (2025). Teacher knows best? The social influence of teachers and peers in high school. J Res Adolesc.

[33] Pouliot A, Vaillancourt R, Stacey D, Suter P (2018). Defining and identifying concepts of medication literacy: an international perspective. Res Social Adm Pharm.

[34] Kopp JP, Jones AT (2020). Impact of item parameter drift on Rasch scale stability in small samples over multiple administrations. Applied Measurement in Education.

[35] Thompson JA, Leyrat C, Fielding KL, Hayes RJ (2022). Cluster randomised trials with a binary outcome and a small number of clusters: comparison of individual and cluster level analysis method. BMC Med Res Methodol.

[36] Qiu H, Cook AJ, Bobb JF (2024). Evaluating tests for cluster-randomized trials with few clusters under generalized linear mixed models with covariate adjustment: a simulation study. Stat Med.

[37] Eysenbach G (2004). Improving the quality of Web surveys: the Checklist for Reporting Results of Internet E-Surveys (CHERRIES). J Med Internet Res.

[38] Polit DF, Beck CT, Owen SV (2007). Is the CVI an acceptable indicator of content validity? Appraisal and recommendations. Res Nurs Health.

[39] Ferreira P, David C, Costescu C, Vera L, Herrera G, Lopes S, Stilwell D, Ferreira A, Domingues D, Brito J, Campos J, Paiva AM, Simão AMV, Heldal I, Stefanut T, Rosan A, Trindade F (2025). Neurodevelopmental disorders: assessing and training working memory. BMC Psychol.

[40] Boone WJ, Staver JR, Yale MS (2014). Rasch Analysis in the Human Sciences.

[41] Ayala A, Pujol R, Forjaz MJ, Abellán A (2019). [Comparison of scaling methods for activities of daily living in older people]. Gac Sanit.

[42] Chang J, Yong L, Yan H, Wang J, Song N (2021). Measurement properties of Canadian Agility and Movement Skill Assessment for children aged 9–12 years using Rasch analysis. Front Public Health.

[43] He Y, Zhuang Y, Feng L, Xu X, Deng Y, Li M, Pang Y, Yao Y, Yu W, Xu Z, Zhou Y, Zhong Y, Li Q, Ye Q, Yuan J, Wen Y, Li J (2025). A novel eye tracking-based gamified assessment of contrast sensitivity function in children: prospective development and reliability study. JMIR Serious Games.

[44] O'Donohoe P, Reasner DS, Kovacs SM, Byrom B, Eremenco S, Barsdorf AI, Arnera V, Coons SJ (2023). Updated recommendations on evidence needed to support measurement comparability among modes of data collection for patient-reported outcome measures: a good practices report of an ISPOR Task Force. Value Health.

[45] Koo TK, Li MY (2016). A guideline of selecting and reporting intraclass correlation coefficients for reliability research. J Chiropr Med.

[46] Martin Bland JM, Altman DG (1986). Statistical methods for assessing agreement between two methods of clinical measurement. Lancet.

[47] McHugh ML (2012). Interrater reliability: the kappa statistic. Biochem Med (Zagreb).

[48] Friehs MA, Schroeder PA, Barlow K, Stein A (2026). Not just childish games: an exploration of different game-based assessments and discussion of gamification in clinical pediatric populations. J Cogn Enhanc.

[49] Husted JA, Cook RJ, Farewell VT, Gladman DD (2000). Methods for assessing responsiveness: a critical review and recommendations. J Clin Epidemiol.

[50] Lakens D (2013). Calculating and reporting effect sizes to facilitate cumulative science: a practical primer for t-tests and ANOVAs. Front Psychol.

[51] Flato UAP, Flato A, Martins IBDT, Simoes Nakano G, Romao JC, Nakano MS, Dos Santos EJB, Villas Boas YDT, Medeiros LE, Rossignoli VG, Cunha GZ, Batista RC, da Silva PGR, Florentino M, Rabelo A, Midega TD, Passos R, Guimaraes H, Kern K (2025). Enhancing equity in schoolchildren's basic life support education in Brazil through serious games: cohort study. JMIR Serious Games.

[52] Rueskov V, Korshøj M, Lund T, Hansen CD, Frausing AE, Larsen CSL, Sommer JN, Mortensen OS (2025). Health literacy among socioeconomically disadvantaged adolescents: a systematic review of interventions in schools. Adolescent Res Rev.

[53] Prokop-Dorner A, Zawisza K, Świątkiewicz-Mośny M, Kobla-Piłat A, Ożegalska-Łukasik N, Ślusarczyk M, Potysz-Rzyman A, M Bala M (2025). Measurement of critical health literacy in primary school pupils: a Polish validation of the Claim Evaluation Tools. BMJ Open.

[54] Paakkari O, Kulmala M, Lyyra N, Torppa M, Mazur J, Boberova Z, Paakkari L (2023). The development and cross-national validation of the short health literacy for school-aged children (HLSAC-5) instrument. Sci Rep.

