# Supplementary material for: Gamified Assessment of Medication Literacy in School-Aged Children: Instrument Development and Validation Study
Source: JMIR Serious Games. 2026 Oct 2;14:e95114. doi: 10.2196/95114 (PMC13675683; doi:10.2196/95114)
Supplement: Supplementary file 1 [file games_v14i1e95114_app1.docx]

**Table S1.** English translations, item justifications, and scoring rules for the gamified assessment tool.

| Item | English translation | Options (score) | Justification | Educational Objective |
| --- | --- | --- | --- | --- |
| Dimension 1: Knowledge  5 items, each with one correct answer, scoring: correct+1, incorrect/“I don’t know”+0 | | | | |
| K1 | What is the main purpose of medicine? | A. Tasty like a snack (0)  B. A lucky charm that prevents all illness (0)  C. Like a reinforcement to help the body fight disease (1)  D. I don’t know (0) | Incorrect options (A, B) reflect common childish misconceptions; correct answer captures scientific concept; “I don’t know” reduces guessing (the same below). | Dietary supplements not medicine substitutes. |
| K2 | Which of the following is not primarily used to treat illness? | A. Dietary supplements (1)  B. Fever reducer (0)  C. I don’t know (0)  D. Eye drops (0) | Incorrect options (B, D) are common pediatric medications that treat illness; correct answer (A) distinguishes supplements from medicines. |  |
| K3 | After completing its task, most of the medicine leaves the body through which fluid? | A. Blood (0)  B. I don’t know (0)  C. Sweat (0)  D. Urine (1) | Incorrect options (A, C) are plausible but incorrect routes of excretion; correct answer (D) reflects knowledge of drug excretion. | No alarm for drug-induced urine discoloration. |
| K4 | Compared with oral medication, what is the biggest drawback of intravenous infusion? | A. I don’t know (0)  B. It is somewhat painful and inconvenient (requires going to hospital) (0)  C. The medicine tastes bitter (0)  D. It may be dangerous (1) | Incorrect answers (B, C) reflect common pediatric experience (pain + inconvenience); correct answer (D) highlights the main disadvantage of IV infusion. | Preference for oral over infusion. |
| K5 | Do most medicines act on only one part of the body? | A. Yes (0)  B. I don’t know (0)  C. No (1) | Incorrect option (A) reflects a common misconception that drugs are site-specific; correct answer (no) indicates understanding that most medications have systemic (non‑selective) effects. | No unilateral discontinuation for side effects. |
| Dimension 2: Attitude  4 items, 5‑point Likert scale, scored 1–5 | | | | |
| A1 | When ill, taking dietary supplements can cure the illness just like medicine. | Strongly disagree (5)  Disagree (4)  I don’t know (3)  Agree (2)  Strongly agree (1) | Disagreement indicates correct understanding. | Dietary supplements not medicine substitutes. |
| A2 | For non‑severe illness, oral medication is preferable to intravenous infusion. | Strongly disagree (1)  Disagree (2)  I don’t know (3)  Agree (4)  Strongly agree (5) | Agreement indicates correct understanding. | Preference for oral over infusion. |
| A3 | If prescribed by a doctor, the medicine should be used even if it has some side effects. | Strongly disagree (1)  Disagree (2)  I don’t know (3)  Agree (4)  Strongly agree (5) | Agreement indicates correct understanding. | No unilateral discontinuation for side effects. |
| A4 | A change in urine color after taking medicine definitely means a side effect has occurred. | Strongly disagree (5)  Disagree (4)  I don’t know (3)  Agree (2)  Strongly agree (1) | Disagreement indicates correct understanding. | No alarm for drug-induced urine discoloration. |
| Dimension 3: Perceived Behavioral Control (PBC)  4 items, multiple answer; scoring: per correct+1, per incorrect–1, “I don’t know”+0; total score mapped to 5 point Likert scale (Mapping: total –2→1, –1→2, 0→3, +1→4, +2→5) | | | | |
| P1 | What are dietary supplements? | A. Nutritious food (1)  B. Can treat diseases (-1)  C. They are medicines (-1)  D. They provide nutrients (1)  E. I don’t know (0) | Correct choices reflect understanding that supplements provide nutrients but do not treat illness; incorrect choices reflect confusion between supplements and medicines. | Dietary supplements not medicine substitutes. |
| P2 | What factors does a doctor consider when choosing between oral and IV medication? | A. Safety (1)  B. Severity/urgency of illness (1)  C. Pain of injection (-1)  D. Bitterness of medicine (-1)  E. I don’t know (0) | Correct choices reflect clinical reasoning; incorrect choices reflect child‑centric concerns (pain, taste). | Preference for oral over infusion. |
| P3 | What do you think about drug side effects? | A. Many drugs have some side effects (1)  B. It means the drug is of poor quality (-1)  C. It only occurs with overdose (-1)  D. Most are mild (1)  E. I don’t know (0) | Correct choices reflect understanding that side effects are common and often mild; incorrect choices reflect fears that side effects indicate poor quality or overdose. | No unilateral discontinuation for side effects. |
| P4 | What do you think about urine colour change after taking medicine? | A. A sign that the medicine is working (-1)  B. Temporary and will disappear (1)  C. Reducing the dose will prevent it (-1)  D. Because the medicine itself has colour (1)  E. I don’t know (0) | Correct choices reflect understanding that harmless urine discoloration is temporary and related to intrinsic drug color; incorrect choices reflect misconceptions about dose reduction or drug action. | No alarm for drug-induced urine discoloration. |
| Dimension 4: Behavioral Intention  4 scenarios, each with 5 options scored 1–5, 5 = most aligned with rational medication literacy | | | | |
| I1 | Lele is ill; doctor suggests pills, parents want IV to get quick recovery. | A. “Doctor said pills are enough, I trust the doctor.” (5)  B. “Maybe pills, but I worry recovery is slow.” (4)  C. “I want IV! Pills are too slow.” (1)  D. “I want IV but I’m afraid of pain.” (2)  E. “I don’t know whom to listen to.” (3) | Option A demonstrates trust in medical advice and resistance to unnecessary IV; option B shows preference for medical advice despite mild concern about recovery speed; option E indicates uncertainty; option D reflects desire for IV tempered by fear of pain; option C prioritizes rapid recovery over medical judgment. | Preference for oral over infusion. |
| I2 | Grandmother suggests dietary supplements instead of medicine. | A. “Whom should I listen to?” (3)  B. “Medicine must be taken; supplements are useless now.” (5)  C. “Grandma loves me, so I’ll stop the medicine.” (1)  D. “Grandma means well, but I shouldn’t stop the medicine.” (4)  E. “I’ll try supplements first, then medicine if not better.” (2) | Option B clearly prioritises prescribed medicine and explicitly states that supplements are useless for treating illness; option D acknowledges grandmother‘s good intention but insists on not stopping the prescribed medicine; option A shows indecision; option E attempts a compromise (supplements first, then medicine) but underestimates the risk; option C completely abandons medicine for supplements. | Dietary supplements not medicine substitutes. |
| I3 | Father worries about side effects on the label. | A. “I’m scared! I won’t take it!” (1)  B. “I don’t know what to do…” (3)  C. “I’m afraid… can we switch to a drug without side effects?” (2)  D. “The doctor prescribed it, it should be fine. If we feel unwell, we’ll tell the doctor.” (5)  E. “I think we should take it, but let’s ask the doctor.” (4) | Option D expresses trust in the doctor and willingness to monitor side effects; option E is similar but less confident; option B shows uncertainty; option C reflects a desire to avoid side effects; option A refuses medicine due to fear of side effects. | No unilateral discontinuation for side effects. |
| I4 | Mother sees urine colour change and suggests stopping the medicine. | A. “I’m worried, but I think we should continue.” (4)  B. “Stop once and observe.” (2)  C. “I don’t know what to do.” (3)  D. “Mom is right, stop immediately.” (1)  E. “Don’t stop yet! We can check the leaflet or ask the doctor.” (5) | Option E not only refuses to stop the medicine but also proactively suggests checking the leaflet or consulting a doctor; option A continues medicine despite worry; option C expresses indecision; option B suggests stopping once to observe; option D immediately agrees to stop. | No alarm for drug-induced urine discoloration. |

*Note*: The “English translation” column is provided solely to help non‑Chinese readers understand the content; they may not fully capture the child‑friendly language and contextual authenticity of the original Chinese items. The “Options (score)” column shows each answer choice with its corresponding score. The “Justification” column explains, for each item, why each incorrect option was chosen (based on common misconceptions identified during cognitive interviews with children) and how the correct and incorrect options discriminate between children with different levels of medication literacy. The “Educational Objective” column indicates the key learning goal associated with each item. Scoring rules are also provided where applicable.

**Table S2.** Badge English translations, design, and rationale for the gamified assessment tool.

| Dimension | Score | Badge name | Design rationale |
| --- | --- | --- | --- |
| Knowledge (max 5) | ≥4 | Wisdom Little Pharmacist | Recognizes solid grasp of medication knowledge. Celebrates mastery and ability to apply learned concepts wisely. |
|  | 2–3 | Knowledge Apprentice | Acknowledges good foundation but still on the learning path. Encourages continued effort and improvement, highlights progress rather than perfection. |
|  | ≤1 | Little Novice in Medication | Emphasizes start and potential. Even if not all answers are correct, it recognizes the efforts of participation and learning basic knowledge. |
| Attitude (max 20) | ≥16 | Pioneer Explorer | Recognizes consistently positive attitudes toward rational medication use. Celebrates proactive stance and willingness to explore safe practices. |
|  | 15–10 | Steady Companion | Acknowledges generally appropriate attitudes, though with some room for further refinement. Reinforces steady progress and encourages continue learning and strengthening health beliefs. |
|  | ≤9 | Thoughtful Observer | Recognizes still developing attitudes. Respects current stage of learning, emphasis thoughtful reflection and openness to new information. |
| PBC (max 20) | ≥16 | Knowledge Sharing Star | Celebrates confidently identifying correct medication‑related information and avoiding common misconceptions. Recognizes ability to not only understand but also potentially share accurate knowledge with others. |
|  | 15–10 | Little Explainer Pro | Acknowledges solid grasp of key medication concepts and distinguishing between correct and incorrect statements most of the time. Encourages continue refining understanding and explaining reasoning. |
|  | ≤9 | Brave Expresser | Rewards participation and expression. Emphasizes that expressing the idea itself is valuable, regardless of its content. |
| Behavioral Intention (max 20) | ≥16 | Health Little Guardian | Rewards recognition and adherence to treatment compliance and integrity.  Celebrates selecting the most appropriate responses in medication‑related dilemmas. |
|  | 15–10 | Professional Good Partner | Rewards healthy behavior patterns that value and rely on professional advice. Acknowledges making appropriate choices but may occasionally need further guidance or show moderate confidence. |
|  | ≤9 | Safety Goalkeeper | Rewards risk awareness and safety vigilance. Recognizes learning to navigate medication dilemmas; responses indicate basic awareness of safety concerns. Values participation and potential to improve. |

*Note:* Each dimension awards a tiered badge based on the total score. Thresholds were chosen to ensure attainability while providing meaningful differentiation. Badge names avoid explicit competitive rankings (e.g., no “Gold/Silver”) to minimise social comparison and anxiety.
